# WBP1 regulates mitochondrial function and ferroptosis to modulate chemoresistance in colorectal cancer

**DOI:** 10.1186/s10020-025-01151-3

**Published:** 2025-03-12

**Authors:** Yang Wang, Dachuan Qi, Guijie Ge, Ning Cao, Xiangdong Liu, Na Zhu, Feng Li, Xiang Huang, Kui Yu, Jinzhou Zheng, Daoheng Wang, Wenyan Yao, Lili Chen, Ziyang Dong

**Affiliations:** 1 Department of General Surgery, Longhua Hospital, Shanghai University of Traditional Chinese Medicine, Shanghai, China; 2 School of Clinical Medicine, Shandong Second Medical University, Weifang, Shandong China; 3 Medical Center of Gastrointestinal Surgery, Weifang People’s Hospital, Weifang, Shandong China; 4 Department of Pharmacy, Weifang People’s Hospital, Weifang, Shandong China

**Keywords:** Colorectal cancer, Mitochondrial function, Chemoresistance, Ferroptosis

## Abstract

Chemoresistance continues to pose a significant challenge in managing colorectal cancer (CRC), resulting in unfavorable outcomes for patients. Recent findings indicate that ferroptosis, an innovative type of regulated cell death, might influence chemoresistance. In this research, we explored how WW domain-binding protein 1 (WBP1) affects mitochondrial function, cell growth, ferroptosis, and chemoresistance in CRC cells. By employing both genetic and pharmacological methods, we found that WBP1 is essential for maintaining mitochondrial respiration in CRC cells. WBP1 depletion impaired mitochondrial function, leading to reduced cell proliferation and increased ferroptosis. Exogenous mitochondria from wild-type cells restored mitochondrial function, cell proliferation, and suppressed ferroptosis in WBP1-deficient cells, indicating that mitochondrial function acts downstream of WBP1. Importantly, we demonstrated that targeting WBP1 or its mediated mitochondrial function sensitized chemoresistant CRC cells to 5-fluorouracil and oxaliplatin by inducing ferroptosis. Furthermore, we analyzed transcriptome data from CRC patients, which indicated that increased WBP1 expression correlated with poor outcomes for patients receiving chemotherapy, thus highlighting the clinical significance of our observations. Collectively, our results pinpoint WBP1 as a significant modulator of mitochondrial function and ferroptosis in CRC cells and imply that targeting WBP1 may represent a viable approach to tackling chemoresistance. These insights offer a deeper understanding of the molecular pathways underlying CRC chemoresistance and may guide the development of new treatment options.

**Supplementary Information:**

The online version contains supplementary material available at 10.1186/s10020-025-01151-3.

## Introduction

Colorectal cancer (CRC) is recognized as one of the most commonly diagnosed malignancies across the globe, holding the position as the third most frequent cancer type, while also being the second leading cause of cancer-related deaths (Hernandez-Alejandro et al. 2022). Although significant advancements have been achieved in therapeutic approaches for CRC, the persistent issue of chemoresistance continues to complicate effective patient management (Khalafizadeh et al. 2024; Rosolen et al. 2023). Chemoresistance, whether it arises intrinsically or is acquired during the course of treatment, contributes to therapeutic failure and subsequently worsens patient outcomes. Thus, elucidating the molecular mechanisms of chemoresistance and identifying novel therapeutic targets is crucial for enhancing CRC patient outcomes.

A growing body of evidence has highlighted the critical role that mitochondrial dysfunction is a key driver of chemoresistance development in various cancer types, particularly in CRC (Wu et al. 2024; Mostafavi et al. 1942). Mitochondria are key organelles responsible for energy production, maintaining redox balance, and regulating cell death pathways (Marzetti et al. 2024; Rickard et al. 2023). Interestingly, it has been observed that an increase in mitochondrial activity, particularly through oxidative phosphorylation, is often associated with higher levels of chemoresistance (Genovese et al. 2021; Giddings et al. 2021; Mukherjee et al. 2023). Cancer cells that display enhanced mitochondrial respiration tend to develop resistance to standard chemotherapeutic drugs such as 5-fluorouracil and oxaliplatin, which are frequently used in CRC treatment (Rainho et al. 2023). This suggests that cancer cells may be adapting to the stress caused by chemotherapy by upregulating mitochondrial functions, thereby promoting drug resistance. As a result, targeting mitochondrial metabolism, specifically by inhibiting oxidative phosphorylation pathways, has been proposed as an effective strategy to enhance chemosensitivity and counteract resistance.

Ferroptosis, a newly characterized form of cell death, is distinct from apoptosis, necrosis, and other forms of cell demise, being primarily driven by iron-dependent lipid peroxidation (Chen et al. 2024; Wang et al. 2024). Recent research has revealed a close link between ferroptosis and mitochondrial function, particularly concerning oxidative stress regulation (Li et al. 2024; Zhou et al. 2024a, b; Adegboro and Afolabi 2024). Mitochondria are major contributors to the production of reactive oxygen species (ROS), and excessive ROS accumulation can lead to lipid peroxidation, which is a hallmark of ferroptosis (Li et al. 2024; Tang et al. 2021). Studies have also demonstrated that inhibiting mitochondrial function can trigger or amplify ferroptosis, providing a potential therapeutic avenue to enhance cancer cell sensitivity to treatment and overcome chemoresistance (Wang et al. 2022; Mao et al. 1947; Huang et al. 2022).

WW domain-binding protein 1 (WBP1) contains two WW domains that facilitate protein-protein interactions, particularly with proteins that possess proline-rich sequences, such as the PPxY motif (Chen et al. 1997; McDonald et al. 2011). Early studies have identified WBP1 as a crucial factor in embryonic development, specifically in the development of bovine embryos to the blastocyst stage (Ortega et al. 2017). While direct studies on WBP1 in cancer are limited, other WW domain-binding proteins, such as WWP1 and WBP2, have been demonstrated to play crucial roles in cancer metabolism. For instance, WWP1 regulates the PTEN-Akt signaling pathway, which is essential for cellular metabolism and growth in cancer cells (Zhang et al. 2015a, b), while WBP2 influences glycolytic enzyme activity and metabolic pathways in glioblastoma (Chen et al. 2018). Given that both embryonic development and cancer progression require precise regulation of cellular metabolism and stress responses, along with the established connections between mitochondrial function, ferroptosis, and chemoresistance in CRC, we hypothesized that WBP1 might serve as a critical mediator of chemoresistance through modulating mitochondrial function and ferroptosis sensitivity. Specifically, we proposed that WBP1 could promote chemoresistance by preserving mitochondrial functionality and inhibiting ferroptosis in CRC cells.

To test this hypothesis, we conducted a comprehensive investigation using both genetic and pharmacological approaches, focusing on three key aspects: (1) the relationship between WBP1 expression and clinical outcomes in CRC patients; (2) the impact of WBP1 on mitochondrial function and cell proliferation; and (3) the potential of targeting WBP1 to overcome chemoresistance through ferroptosis induction. Our findings not only provide new insights into the molecular mechanisms driving chemoresistance in CRC but also identify WBP1 as a promising therapeutic target for chemoresistant CRC treatment through the modulation of mitochondrial function and ferroptosis.

## Materials and methods

### Cell models and culture conditions

The human CRC lines HCT116 (RRID: CVCL_0291) and SW480 (RRID: CVCL_0546) were purchased from ATCC. HCT116 cells were originally isolated from a male patient with Duke’s stage D colorectal carcinoma and represent a microsatellite instability-high (MSI-H) cell line with metastatic potential (Ahmed et al. 2013; Mooi et al. 2018). SW480 cells were derived from the large intestine of a Dukes B colorectal cancer patient, representing a moderately metastatic microsatellite stable (MSS) cell line (Ahmed et al. 2013; Mooi et al. 2018). Both cell lines are well-characterized models for studying CRC progression and drug resistance. Cells were maintained in DMEM media enriched with 10% FBS and 1% penicillin/streptomycin (all from Gibco) in standard culture conditions (37 °C, 5% CO2). Authentication of cell lines was performed using STR profiling within six months before experimentation. Regular mycoplasma testing was conducted using LookOut^®^ mycoplasma qPCR detection kit (Sigma).

### Immunofluorescence staining

Cells were plated on sterile glass coverslips and allowed to adhere overnight. Mitochondria were labeled using MitoTracker Red CMXRos (500 nM) for 30 min at 37 °C. Cells were then fixed with 4% paraformaldehyde for 15 min, followed by permeabilization with 0.1% Triton X-100 at room temperature for 10 min. After blocking with 5% bovine serum albumin (BSA) for 1 h, primary antibodies against WBP1 (1:100, Sigma, cat. #: HPA057067) were applied overnight at 4 °C. The following day, cells were rinsed with PBS and incubated with Alexa Fluor 488-conjugated secondary antibodies (1:500, Invitrogen) for 1 h at room temperature. Nuclei were stained with DAPI (1 µg/mL) for 20 min, and coverslips were mounted on slides using ProLong Gold Antifade Mountant (Invitrogen). Images were captured using a Zeiss LSM 800 confocal microscope.

### WBP1 knockout cell line generation

CRISPR-Cas9 technology was employed to generate WBP1 knockout lines. Guide RNAs (TCATCAGGAGGGTGAGCCCG and TGTACCGCAGATCTTTCCCA) targeting WBP1 exon 4 were cloned into pSpCas9(BB)-2 A-Puro (PX459) V2.0 (Addgene). Lentiviral particles were generated in HEK293T cells through co-transfection of gRNA constructs with psPAX2 and pMD2.G using Lipofectamine 2000. Target cells underwent lentiviral transduction followed by puromycin selection (2 µg/mL, 7 days). Single-cell isolation yielded knockout clones.

### Lentiviral transduction for WBP1 overexpression

For WBP1 overexpression, human WBP1 cDNA was inserted into pLenti-CMV-Puro DEST vector. Viral production and cell transduction procedures followed the same protocol as knockout generation.

### Cell proliferation assay

Cell proliferation was measured using the sulforhodamine B (SRB) assay. Cells were seeded at a density of 2,000 cells/well in 96-well plates. After overnight adhesion, cells were fixed with 10% trichloroacetic acid (TCA) at 4 °C for 1 h. Plates were washed with water, stained with 0.4% SRB solution for 30 min at room temperature, and then rinsed with 1% acetic acid. After air-drying, bound SRB dye was solubilized with 10 mM Tris base (pH 10.5), and absorbance was measured at 565 nm using a microplate reader (BioTek).

### TCGA data acquisition and analysis

RNA sequencing data were downloaded from TCGA-COAD project (https://portal.gdc.cancer.gov/projects/TCGA-COAD, Project ID: TCGA-COAD, dbGaP Study Accession: phs000178) using the ‘TCGAbiolinks’ R package. The dataset included primary colorectal adenocarcinoma (n = 432) and normal tissues (n = 41). Raw count data were normalized using ‘DESeq2’ R package. Clinical information was obtained from the associated clinical data files. Data processing and statistical analyses were performed using R software. WBP1 expression differences between normal and tumor tissues were visualized using boxplots and analyzed using Wilcoxon rank-sum test. Survival analysis was conducted using the ‘survival’ R package.

### Measurement of oxygen consumption rate (OCR)

Mitochondrial respiration was evaluated by measuring the oxygen consumption rate (OCR) using a Seahorse XFe96 Analyzer (Agilent). Cells were seeded in XFe96 microplates at 20,000 cells/well and allowed to adhere overnight. Cells were washed the next day using Seahorse XF Base Medium (containing glucose [10 mM], L-glutamine [2 mM], and sodium pyruvate [1 mM], pH 7.4). After 1-hour incubation (37 °C, non-CO₂ incubator), baseline OCR and responses to oligomycin (1 µM), FCCP (0.5 µM), and rotenone/antimycin A (0.5 µM each) were measured. Data were normalized to protein concentration. Basal OCR was calculated by subtracting the non-mitochondrial OCR (minimum rate after rotenone/antimycin A injection) from the baseline OCR (last rate before oligomycin injection). ATP-linked OCR was calculated by subtracting the oligomycin-insensitive OCR (minimum rate after oligomycin injection) from the basal OCR. Maximal OCR was calculated by subtracting the non-mitochondrial OCR from the maximum rate after FCCP injection.

### Gene expression analysis

RNA isolation utilized the RNeasy Mini Kit (Qiagen). cDNA synthesis was performed using High-Capacity cDNA Reverse Transcription Kit (Applied Biosystems) with 1 µg RNA input. Real-time PCR employed PowerUp SYBR Green chemistry on an Applied Biosystems 7500 Real-Time PCR system. Relative expression was calculated using the 2-ΔΔCt method with TUBA1A normalization. The primer sequences used were as follows: MT-ND1 (forward: 5’- TCCTACTCCTCATTGTACCCA − 3’, reverse: 5’- TTTCGTTCGGTAAGCATTAGG − 3’), MT-CO1 (forward: 5’- ATATTTCACCTCCGCTACCA − 3’), reverse: 5’- TCAGCTAAATACTTTGACGCC − 3’), GPX4 (forward: 5’- ATTGGTCGGCTGGACGAG-3’, reverse: 5’- CTTCAGTAGGCGGCAAAGGC-3’), FTH1 (forward: 5’- ACTACCACCAGGACTCAGAGG-3’, reverse: 5’- CAGGTAAACGTAGGAGGCGT-3’), WBP1 (forward: 5’- GGTGTTTCCTCCCACCAGAG-3’, reverse: 5’- GGACAAGGGCAGAGCTCAAT − 3’), and TUBA1A (forward: 5’- GAAGCAGCAACCATGCGTGA-3’, reverse: 5’- CCTCCCCCAATGGTCTTGTC-3’).

### Western blot analysis

Cells were lysed using RIPA buffer (Beyotime, P0013B) that included a protease inhibitor cocktail (Roche, 04693159001). The protein concentrations were quantified with the BCA protein assay kit (Thermo Fisher Scientific, 23225). An equivalent quantity of protein (30 µg) was resolved by SDS-PAGE and transferred to PVDF membranes (Millipore, IPVH00010). Following a 1-hour blocking step at room temperature with 5% non-fat milk, the membranes were incubated overnight at 4 °C with primary antibodies specific to MT-ND1 (Abcam, ab181848, 1:1000), MT-CO1 (Abcam, ab14705, 1:1000), GPX4 (Cell Signaling Technology, #52455, 1:1000), FTH1 (Cell Signaling Technology, #4393, 1:1000), WBP1 (Sigma, SAB4301812, 1:1000), and TUBA1A (Sigma, T9026, 1:5000). Afterward, the membranes were treated with HRP-conjugated anti-rabbit IgG (Cell Signaling Technology, #7074, 1:5000) or anti-mouse IgG (Cell Signaling Technology, #7076, 1:5000) for 1 h at room temperature. Visualization of protein bands was achieved using an enhanced chemiluminescence detection system (Millipore, WBKLS0500).

### Mitochondrial membrane potential assay

The assessment of mitochondrial membrane potential utilized the JC-1 Mitochondrial Membrane Potential Assay Kit (ab113850, Abcam). Cells were treated with dilution buffer and then incubated with JC-1 dye solution for 10 min at 37 °C. After a wash with dilution buffer, fluorescence was recorded using a fluorescence microplate reader, with excitation wavelengths of 535 nm for J-aggregates (red) and 475 nm for both J-aggregates and monomers (green). The red to green fluorescence ratio was calculated to determine alterations in mitochondrial membrane potential.

### Cellular ROS measurement

Intracellular ROS levels were measured using the Cellular ROS Assay Kit Deep Red (ab186029, Abcam) following the manufacturer’s protocol. Cells were seeded in 96-well plates and cultured overnight. The cells were then incubated with ROS Deep Red dye at 37 °C for 1 h in the dark. The fluorescence intensity was then measured using a fluorescence microplate reader with excitation/emission at 650/675 nm. The relative ROS levels were normalized to the WT group.

### Lipid peroxidation (LPO) and iron assay

LPO levels was measured using the Lipid Peroxidation (MDA) Assay Kit (Abcam) according to the manufacturer’s instructions. Following sample preparation, the fluorescence intensity of MDA-TBA adducts was measured using a fluorescence microplate reader (excitation/emission: 532/553 nm). Total iron content was determined using the Iron Assay Kit (Abcam). The absorbance was assessed at 593 nm using a microplate reader.

### Calculation of ferroptosis score

The ferroptosis score calculation was performed according to a previously established and validated methodology in cancer research (He et al. 2022). Briefly, ferroptosis-related genes (FRGs) were obtained from the FerrDB database and categorized into two functional groups: positive core components (pro-FRGs: ACSL4, ALOX5, NOX4, TF, ATG3, MAPK9, ATG5, BECN1, PRKAA1, SCP2, BACH1, HIF1A, MTDH, PI3KCA, SOCS1, TLR4, ATM, IFNG, ZEB1, FLT3, ATF3, MYB, SLC38A1) and negative core components (anti-FRGs: SLC3A2, CBS, NQO1, PROM2, HSPB1, VDAC2, NF2, FH, BRD4). The ferroptosis score was computed employing the single-sample Gene Set Enrichment Analysis (ssGSEA) algorithm. Specifically, enrichment scores were first computed separately for both pro-FRGs and anti-FRGs gene sets. The final ferroptosis score for each sample was then determined by subtracting the anti-FRGs enrichment score from the pro-FRGs enrichment score. Based on the median WBP1 expression value, samples were divided into high (*n* = 216) and low (*n* = 216) WBP1 expression groups for comparison.

### Mitochondrial extraction and supplementation

Fresh mitochondria were isolated from HCT116 and SW480 cells using a commercial isolation kit (Mitochondria Isolation Kit for Cultured Cells, Thermo Fisher Scientific), following the manufacturer’s protocol. The protein concentration of the isolated mitochondria was evaluated through the BCA protein quantification assay (Pierce). For the experiments involving mitochondrial supplementation, WBP1 knockout HCT116 and SW480 cells were cultivated in 6-well culture plates until they reached 60–80% confluence. The isolated mitochondrial fraction (100 µg total protein per well) from corresponding wild-type cells was supplemented directly into the culture medium of WBP1 knockout recipient cells. Following a 24-hour incubation period under standard culture conditions (37 °C, 5% CO2), the cells were collected for downstream experimental analyses.

### Generation of chemoresistant cell lines

The establishment of chemoresistant cell lines was performed according to previously reported methods with appropriate modifications (Boyer et al. 2004; Wang et al. 2020a, b; Xu et al. 2019; Ye et al. 2017). Briefly, SW480 cells were continuously exposed to gradually increasing concentrations of 5-FU or oxaliplatin for 8 months. The initial concentrations were 1 µM for 5-FU and 0.2 µM for oxaliplatin. Drug concentrations were incrementally increased by 20% every two weeks if cells showed stable growth. The final concentrations reached 8 µM for 5-FU and 1.5 µM for oxaliplatin. The drug resistance was verified using cell viability assays. The established resistant cell lines (designated as 5FU-R and oxaliplatin-R) were maintained in medium containing 2 µM 5-FU or 0.5 µM oxaliplatin, respectively. Drug resistance was regularly verified by dose-response assays.

### Dose-response curves and cell viability assay

SW480 cells, as well as their chemoresistant derivatives, were seeded at a density of 3,000 cells per well in 96-well plates and allowed to adhere overnight. Cells were then treated with increasing concentrations of 5-FU or oxaliplatin for 72 h. Cell viability was measured using the SRB assay, as previously described. A Four-Parameter Logistic (4PL) model was applied for dose-response curve analysis, and the half-maximal inhibitory concentration (IC50) values were calculated.

### Statistical analysis

Quantitative results are expressed as the mean with standard deviation of at least three independent experiments. Comparisons between experimental groups were performed by Student’s t-test. Analysis of patient survival was conducted using the Kaplan-Meier method, with differences between groups assessed by log-rank statistics. Differences were considered statistically meaningful when the probability value (p) was less than 0.05.

## Results

### WBP1 is found within the mitochondria of CRC cells and plays a role in regulating both cell proliferation and mitochondrial respiration

**Fig. 1:**
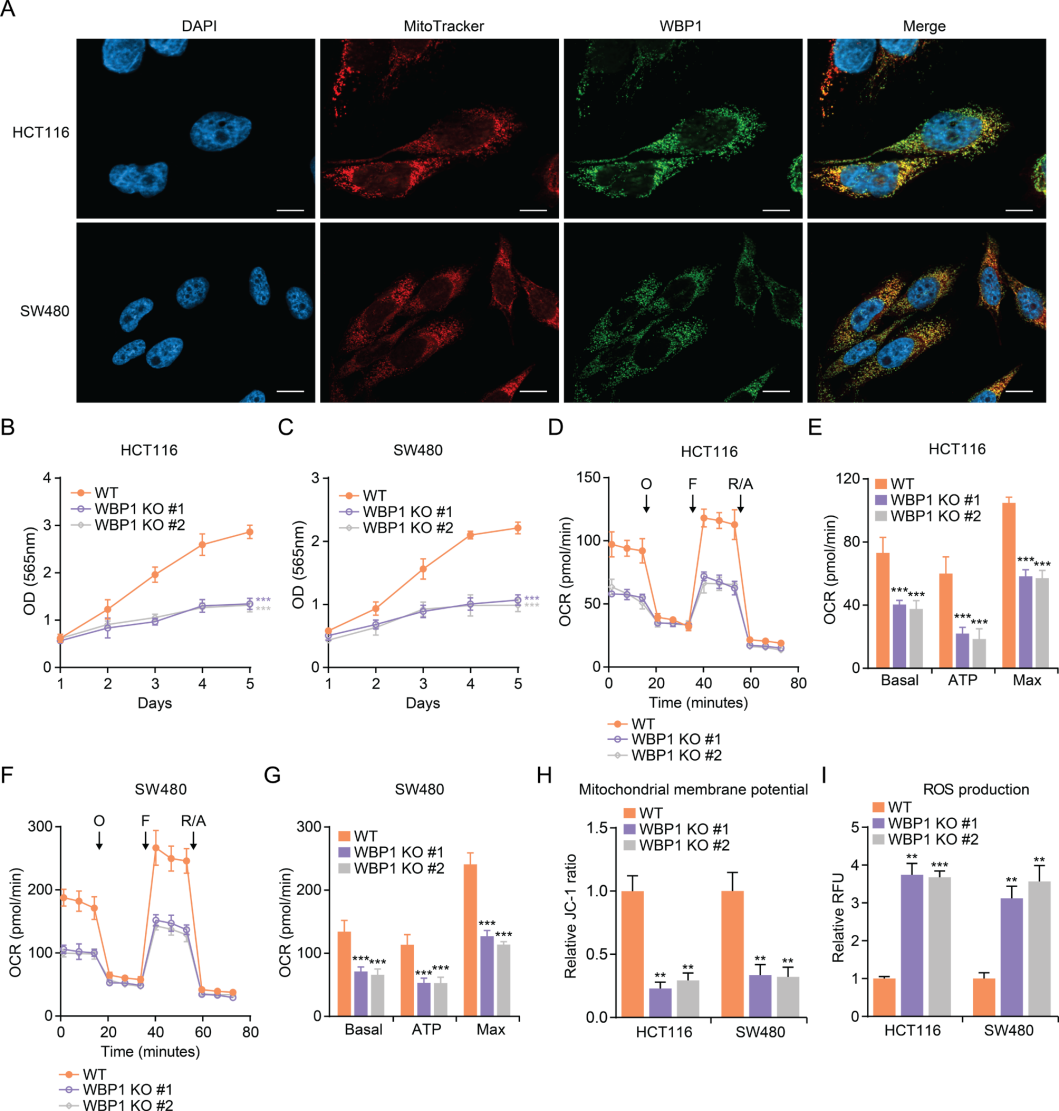
The presence of WBP1 in CRC cell mitochondria is vital for maintaining cellular proliferation and respiratory function. (**A**) Representative immunofluorescence images showing the subcellular localization of WBP1 in wild-type (WT), WBP1 knockout (KO) #1, and WBP1 KO #2 HCT116 and SW480 cells. Cells were stained with MitoTracker (red) to label mitochondria, DAPI (blue) to label nuclei, and WBP1 (green). Scale bar: 10 μm. (**B**-**C**) Cell proliferation of WT, WBP1 KO #1, and WBP1 KO #2 HCT116 (**B**) and SW480 (**C**) cells assessed by sulforhodamine B (SRB) assay (*n* = 3). (**D**-**G**) Quantification of oxygen consumption rate (OCR) in WT, WBP1 KO #1, and WBP1 KO #2 HCT116 (**D**, **E**) and SW480 (**F**, **G**) cells measured by Seahorse XF analyzer (*n* = 6). OCR was measured under basal conditions and in response to sequential addition of oligomycin (**O**), FCCP (**F**), and rotenone/antimycin A (R/A). (H-I) Relative mitochondrial membrane potential measured by JC-1 staining (H) and relative ROS levels measured by a commercial Cellular ROS Assay Kit (**I**) in WT, WBP1 KO #1, and WBP1 KO #2 HCT116 and SW480 cells (*n* = 3). Data are shown as mean ± SD with statistical significance determined by Student’s t-test (***p < 0.001)

To explore WBP1’s function in CRC, we initially assessed its localization within the cell using immunofluorescence staining. In both HCT116 and SW480 CRC cell lines, WBP1 colocalized with the mitochondrial marker MitoTracker, indicating that WBP1 is predominantly localized in the mitochondria (Fig. 1A). To further explore the function of WBP1, we generated WBP1 knockout (KO) cell lines using CRISPR-Cas9 and assessed the effect of WBP1 depletion on cell proliferation using the sulforhodamine B (SRB) assay. WBP1 KO significantly impaired cell proliferation in both HCT116 (Fig. 1B) and SW480 (Fig. 1C) cells in comparison with wild-type (WT) cells. As WBP1 is localized in the mitochondria, we further investigated whether WBP1 depletion affects mitochondrial respiration. To this end, we measured the oxygen consumption rate (OCR) in WT and WBP1 KO cells. In both HCT116 (Fig. 1D and E) and SW480 (Fig. 1F and G) cells, WBP1 KO led to a marked reduction in basal, ATP-linked, and maximal OCR compared to WT cells. Consistently, WBP1 KO markedly decreased the expression of mitochondrial respiratory chain components MT-ND1 and MT-CO1 at both mRNA and protein levels (Figures S1A-D). Additionally, WBP1 KO cells showed a notable reduction in mitochondrial membrane potential (Fig. 1H) and increased intracellular ROS levels (Fig. 1I) compared to WT cells. These results suggest that WBP1 is essential for maintaining mitochondrial respiration and cell proliferation in CRC cells.

### WBP1 knockout triggers ferroptosis in CRC cells

**Fig. 2:**
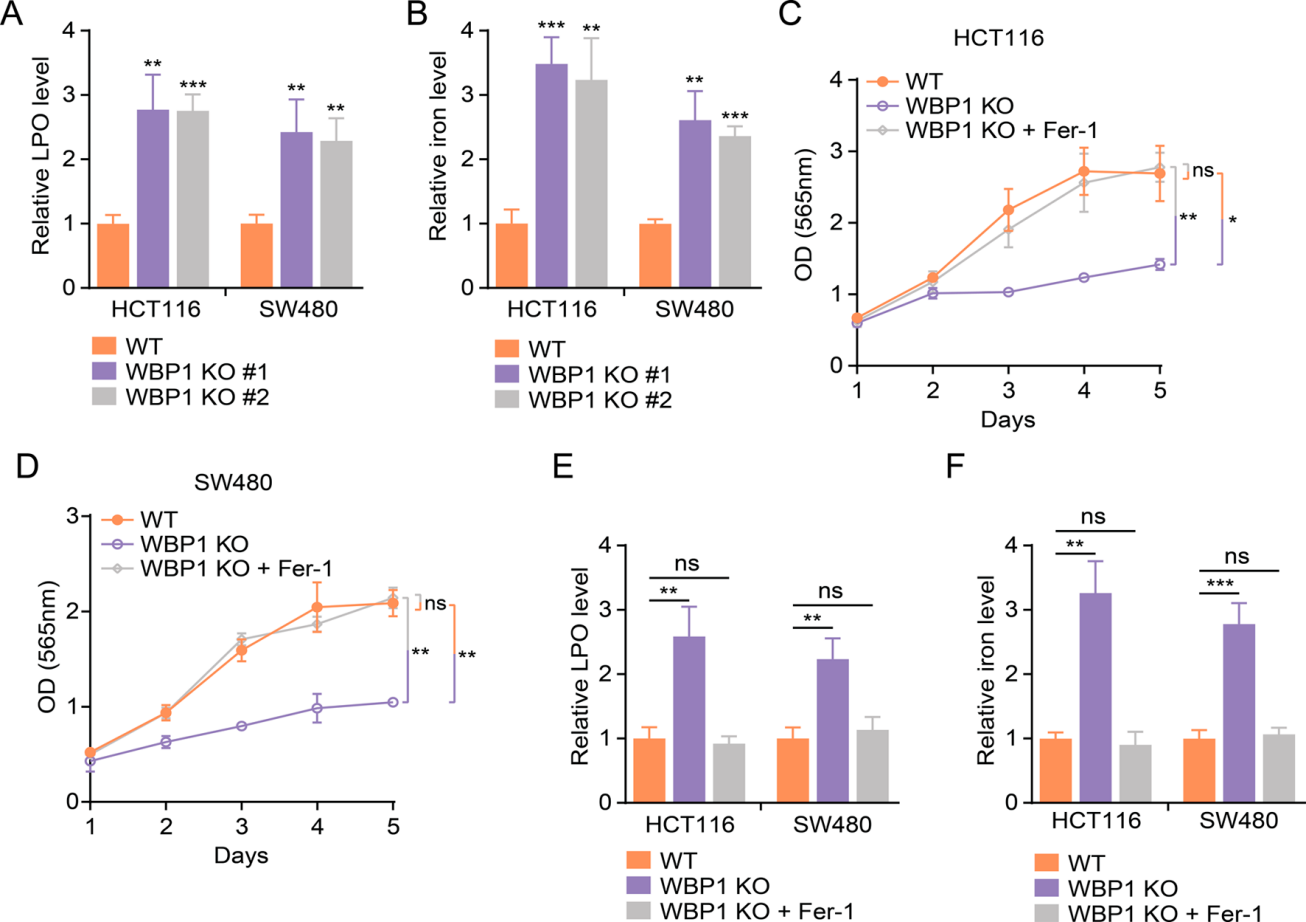
WBP1 knockout induces ferroptosis in CRC cells. (**A**-**B**) Relative lipid peroxidation (LPO) (**A**) and iron levels (**B**) in WT and WBP1 KO HCT116 and SW480 cells (*n* = 4; WBP1 KO indicates WBP1 KO #1 clone). (**C**-**D**) Cell proliferation of WT, WBP1 KO, and WBP1 KO cells exposed to 2 µM Ferrostatin-1 (Fer-1) in HCT116 (C) and SW480 (D) cells assessed by SRB assay (*n* = 3; WBP1 KO indicates WBP1 KO #1 clone). (**E**-**F**) Relative LPO (**E**) and iron levels (**F**) in WT, WBP1 KO, and WBP1 KO cells treated with 2 µM Fer-1 in HCT116 and SW480 cells (*n* = 4; WBP1 KO indicates WBP1 KO #1 clone). Results are expressed as mean ± SD. Statistical analysis was performed using Student’s t-test (*p < 0.05, **p < 0.01, ***p < 0.001, ns: not significant)

Given the critical role of mitochondria in regulating ferroptosis (Li et al. 2024; Zhou et al. 2024a, b; Adegboro and Afolabi 2024), a form of iron-dependent cell death, we hypothesized that WBP1 depletion might induce ferroptosis in CRC cells. To test this hypothesis, we measured lipid peroxidation (LPO) and iron levels, two hallmarks of ferroptosis (Rochette et al. 2022; Krusenstiern et al. 2023; Chen et al. 2021), in WT and WBP1 KO cells. We found that WBP1 KO significantly increased LPO (Fig. 2A) and iron levels (Fig. 2B) in CRC cells. Supporting the occurrence of ferroptosis, WBP1 KO significantly decreased the expression of key ferroptosis suppressors GPX4 and FTH1 at both mRNA and protein levels (Figures S2A-D). To further confirm that WBP1 depletion induces ferroptosis, we treated WBP1 KO cells with ferrostatin-1 (Fer-1), a specific inhibitor of ferroptosis (Ma et al. 2022; Scarpellini et al. 2023; Miotto et al. 2020) and observed that Fer-1 treatment significantly rescued the impaired cell proliferation in WBP1 KO CRC cells (Fig. 2C and D). Moreover, Fer-1 treatment abolished the increased LPO (Fig. 2E) and iron levels (Fig. 2F) in WBP1 KO cells. Together, these results indicate that WBP1 depletion induces ferroptosis in CRC cells.

### High WBP1 expression correlates with poor survival and reduced ferroptosis score in CRC patients

**Fig. 3:**
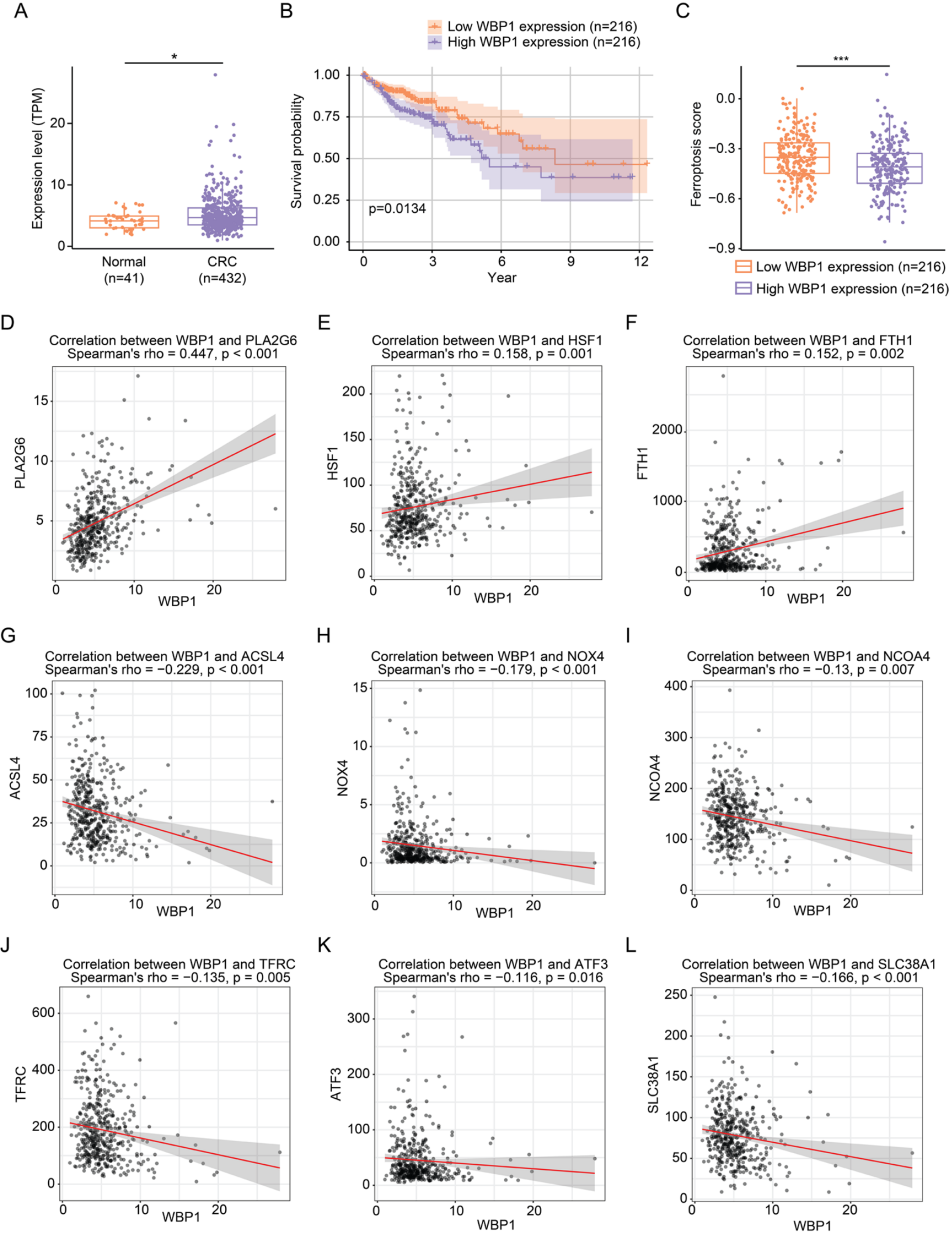
High WBP1 expression is associated with poor survival and lower ferroptosis score in CRC patients. (**A**) WBP1 expression in normal colorectal tissues (*n* = 41) and CRC tissues (*n* = 432) from TCGA dataset (TCGA-COAD). *P < 0.05, determined by Wilcoxon rank-sum test. (**B**) Kaplan-Meier analysis of overall survival in CRC patients stratified by WBP1 expression levels. The median expression value of WBP1 served as the cutoff to classify patients into high and low expression groups. The p-value was calculated using the log-rank test. (**C**) Ferroptosis score in CRC patients with low and high WBP1 expression was calculated based on a previously published method ^28^. ***P < 0.001, determined by Student’s t-test. (**D**-**F**) Correlation analysis between WBP1 expression and ferroptosis suppressors PLA2G6 (D), HSF1 (**E**), and FTH1 (**F**) in CRC tissues from TCGA dataset (TCGA-COAD). (**G**-**L**) Correlation analysis between WBP1 expression and ferroptosis drivers ACSL4 (**G**), NOX4 (**H**), NCOA4 (**I**), TFRC (**J**), ATF3 (**K**), and SLC38A1 (**L**) in CRC tissues from TCGA dataset (TCGA-COAD). Spearman’s correlation coefficient (rho) and p-values are shown in each plot

To assess WBP1’s clinical relevance, we first analyzed WBP1 expression levels between normal colorectal tissues and CRC tissues using The Cancer Genome Atlas (TCGA) dataset. WBP1 was significantly upregulated in CRC tissues compared to normal tissues, suggesting it might serve as a potential biomarker for CRC (Fig. 3A). We then evaluated patient survival data against gene expression profiles from The Cancer Genome Atlas (TCGA) colorectal cancer dataset. Survival analysis demonstrated that high WBP1 expression correlated with poor prognosis, as evidenced by reduced overall survival in Kaplan-Meier plots (Fig. 3B). As ferroptosis is a tumor-suppressive mechanism, we next calculated the ferroptosis score in CRC patients with low and high WBP1 expression based on a previously published method (He et al. 2022). Patients exhibiting elevated WBP1 expression had a markedly lower ferroptosis score compared to those with lower WBP1 levels (Fig. 3C). Overall, these findings indicate that increased WBP1 expression is linked to worse prognosis and diminished ferroptosis in CRC patients.

To further understand how WBP1 regulates ferroptosis, we analyzed the expression correlation between WBP1 and well-established ferroptosis-related genes. Notably, WBP1 expression showed significant positive correlations with ferroptosis suppressors including PLA2G6 (Beharier et al. 2020; Sun et al. 2021), HSF1 (Jia et al.2023a), and FTH1 (He et al. 2023; Tian et al. 2020) (Fig. 3D-F). Conversely, WBP1 exhibited significant negative correlations with several key ferroptosis drivers, including ACSL4 (Doll et al. 2017; Jia et al. 2023b), NOX4 (Maimaiti et al. 46; Park et al. 2021), NCOA4 (Santana-Codina et al. 2021), TFRC (Yi et al. 2022; Zhou et al. 2024a, b), ATF3 (Li et al. 2022; Wang et al. 2020), and SLC38A1 (Li et al. 2023; Luo et al. 2023) (Fig. 3G-L). These correlation patterns provide additional evidence supporting WBP1’s role in ferroptosis regulation and explain the reduced ferroptosis score observed in patients with high WBP1 expression.

### Exogenous mitochondria restore mitochondrial respiration, cell proliferation, and suppress ferroptosis in WBP1-deficient CRC cells

**Fig. 4:**
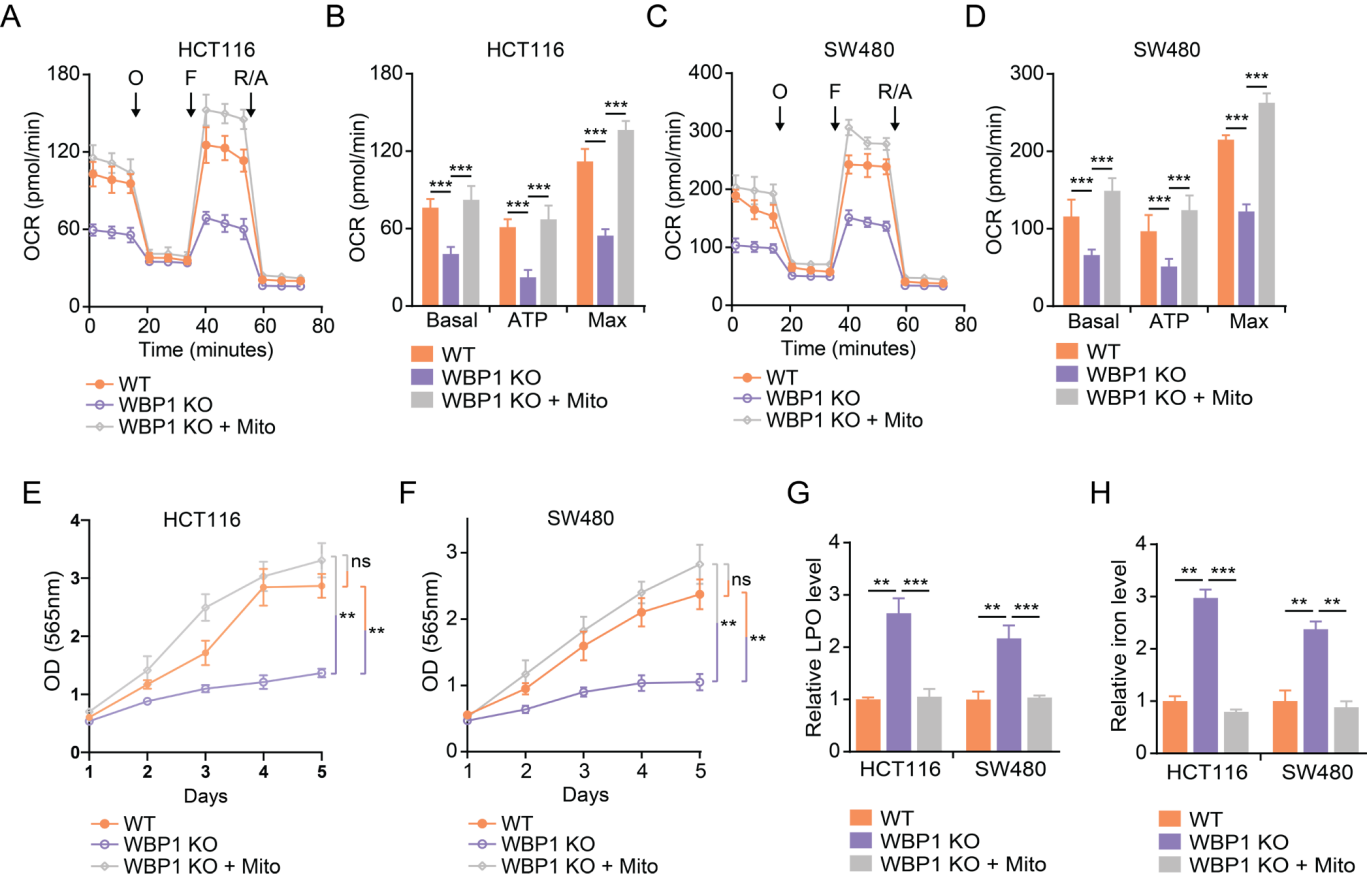
Exogenous mitochondria from WT cells rescue the phenotypes induced by WBP1 knockout in CRC cells. (**A**-**D**) OCR measurements comparing WT cells, WBP1 KO cells, and WBP1 KO cells supplemented with WT mitochondria in (**A**, **B**) HCT116 and (**C**, **D**) SW480 cell lines by Seahorse XF analyzer (WBP1 KO indicates WBP1 KO #1 clone). (**B**, **D**) Quantification of basal, ATP-linked, and maximal OCR based on the data from **A** and **C**, respectively. (**E**, **F**) Cell proliferation of WT, WBP1 KO, and WBP1 KO cells following WT mitochondrial transfer in HCT116 (**E**) and SW480 (**F**) cells assessed by SRB assay (WBP1 KO indicates WBP1 KO #1 clone). (**G**, **H**) Relative LPO (**G**) and iron levels (H) in WT, WBP1 KO, and WBP1 KO HCT116 and SW480 cells treated with mitochondria isolated from WT cells in HCT116 and SW480 cells, respectively (WBP1 KO indicates WBP1 KO #1 clone). Data are presented as mean ± SD (*n* = 6 for **A**-**D**, *n* = 3 for **E**-**F**, *n* = 4 for **G**-**H**). Student’s t-test was used for statistical analysis (**p < 0.01, ***p < 0.001)

To further explore whether the defects in mitochondrial respiration are responsible for the phenotypes seen in WBP1 knockout cells, we extracted mitochondria from wild-type cells and transferred them into WBP1 knockout cells. OCR analysis revealed that introducing exogenous mitochondria from wild-type cells led to a significant increase in basal, ATP-associated, and maximal OCR in WBP1 KO CRC cells (Fig. 4A-D). Moreover, the exogenous mitochondria also rescued the impaired cell proliferation in WBP1 KO CRC cells (Fig. 4E and F). In addition, the increased LPO (Fig. 4G) and iron levels (Fig. 4H) in WBP1 KO cells were abolished by the introduction of exogenous mitochondria. These results suggest that the impaired mitochondrial respiration is responsible for the ferroptosis and growth inhibition induced by WBP1 depletion in CRC cells, and restoring mitochondrial function can suppress ferroptosis and rescue cell proliferation in WBP1-deficient cells.

### Inhibition of mitochondrial respiration by rotenone recapitulates WBP1 knockout phenotypes and cannot be rescued by WBP1 overexpression

**Fig. 5:**
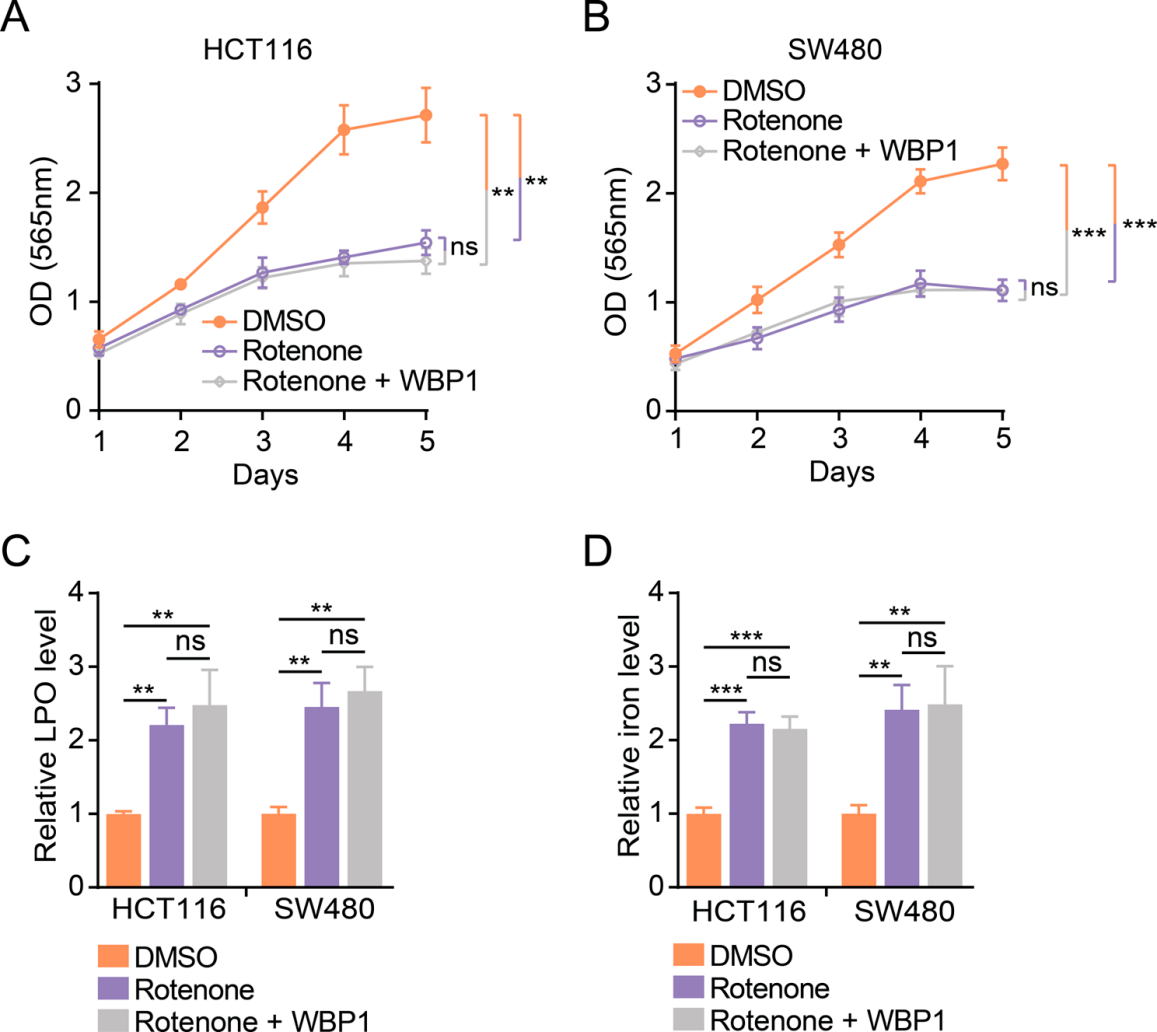
Rotenone treatment mimics WBP1 knockout phenotypes, which cannot be rescued by WBP1 overexpression in CRC cells. (**A**, **B**) Cell proliferation of HCT116 (**A**) and SW480 (**B**) cells treated with DMSO, rotenone, or rotenone in WBP1-overexpressing cells assessed by SRB assay (*n* = 3). Rotenone was used at a concentration of 100 nM for HCT116 cells and 200 nM for SW480 cells. (**C**, **D**) Relative LPO (**C**) and iron levels (**D**) in HCT116 and SW480 cells treated with DMSO, rotenone, or rotenone in WBP1-overexpressing cells (*n* = 4). Rotenone was used at a concentration of 100 nM for HCT116 cells and 200 nM for SW480 cells. Results are shown as mean ± SD. Statistical analysis was performed using Student’s t-test (**p < 0.01, ***p < 0.001, ns: not significant)

To further confirm the role of mitochondrial respiration in mediating the effects of WBP1, we treated CRC cells with rotenone, a well-known inhibitor of mitochondrial function that blocks electron transfer from iron-sulfur centers in mitochondrial complex I to ubiquinone (Li et al. 2003; Pereira et al. 2023; Heinz et al. 2017). Based on previous literature (Altilia et al. 2012; Thomas et al. 2019; Shi et al. 2021) and our preliminary experiments, rotenone was used at optimized concentrations of 100 nM for HCT116 cells and 200 nM for SW480 cells. Rotenone treatment significantly impaired cell proliferation in CRC cells (Fig. 5A and B), mimicking the effects of WBP1 knockout. Moreover, rotenone treatment increased LPO (Fig. 5C) and iron levels (Fig. 5D) in CRC cells, similar to the effects of WBP1 depletion. Interestingly, WBP1 overexpression could not rescue the impaired cell proliferation (Fig. 5A-B, S3A-B) or increased LPO (Fig. 5C) and iron levels (Fig. 5D) induced by rotenone treatment. These results suggest that mitochondrial function acts downstream of WBP1, and WBP1 exerts its effects by regulating mitochondrial function. Therefore, when mitochondrial function is inhibited by rotenone, overexpression of WBP1 cannot rescue the phenotypes, as its downstream effector is already compromised. This indicates that WBP1 plays a role in maintaining mitochondrial function, which is essential for its tumor-promoting effects in CRC cells.

### WBP1 depletion or mitochondrial function Inhibition sensitizes chemoresistant CRC cells to chemotherapy by inducing ferroptosis

**Fig. 6:**
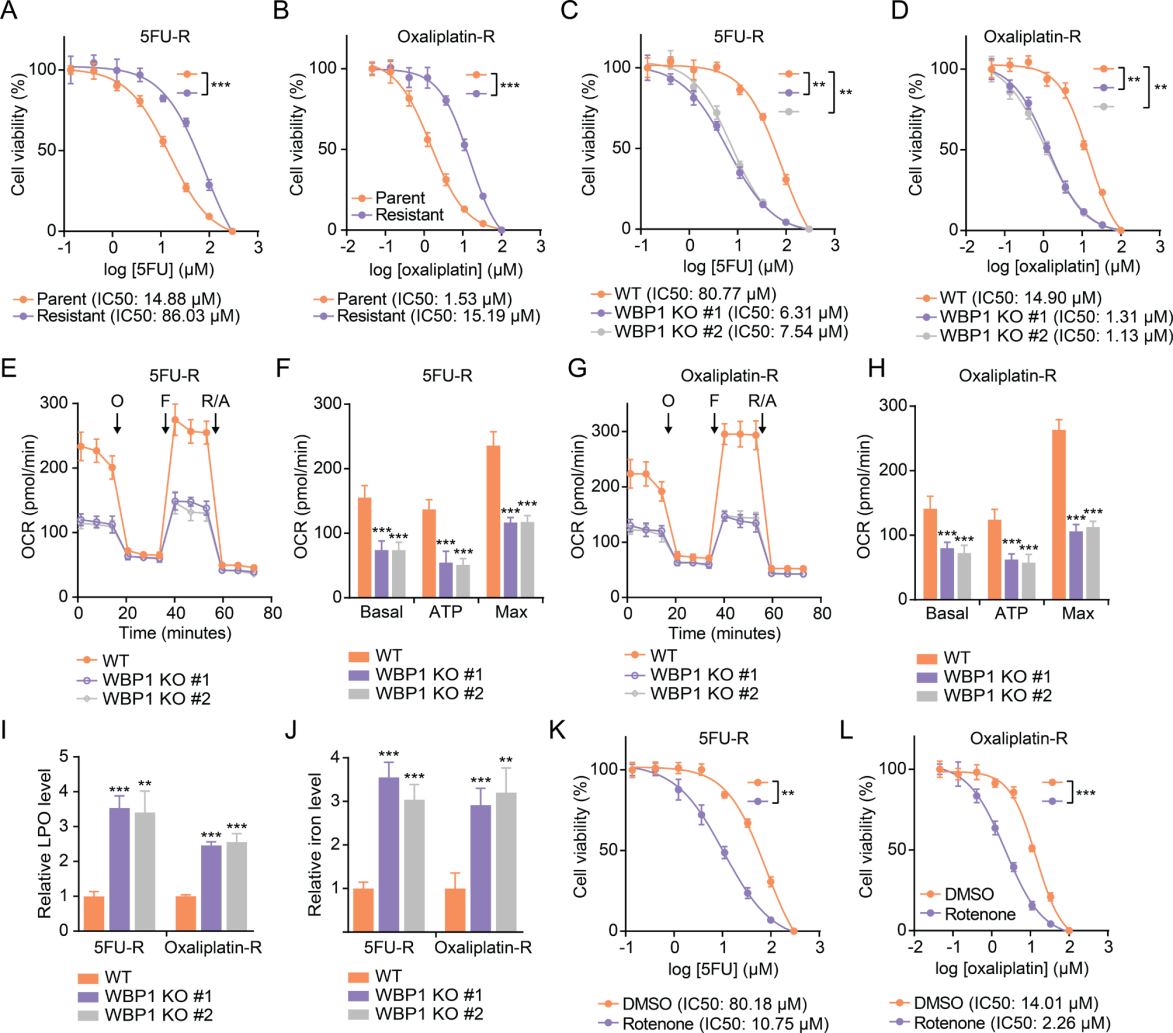
Targeting WBP1 overcomes CRC chemoresistance through disruption of mitochondrial respiration and ferroptosis induction. (**A**, **B**) Cell viability of parental and 5FU-resistant (5FU-R) (**A**) or oxaliplatin-resistant (oxaliplatin-R) (**B**) SW480 cells treated with different concentrations of 5FU (**A**) or oxaliplatin (**B**) for 72 h, assessed by SRB assay. (**C**, **D**) Cell viability of WT and WBP1 KO 5FU-R (**C**) and oxaliplatin-R (**D**) SW480 cells treated with different concentrations of 5FU (**C**) or oxaliplatin (**D**) for 72 h, assessed by SRB assay. (**E**-**H**) OCR of WT and WBP1 KO 5FU-R (**E**, **F**) and oxaliplatin-R (**G**, **H**) SW480 cells measured by Seahorse XF analyzer. (F, H) Quantification of basal, ATP-linked, and maximal OCR based on the profiles from **E** and **G**, respectively. (**I**, **J**) Relative LPO levels (**I**) and iron levels (**J**) in WT and WBP1 KO 5FU-R and oxaliplatin-R SW480 cells. (**K**, **L**) Cell viability of DMSO- or 200 nM rotenone-treated 5FU-R (K) and oxaliplatin-R (L) SW480 cells treated with different concentrations of 5FU (**K**) or oxaliplatin (**L**) for 72 h, assessed by SRB assay. Data are presented as mean ± SD (*n* = 3 for **A**-**D**, K, L; *n* = 6 for **E**-**H**; *n* = 4 for **I**, **J**). **P < 0.01, ***P < 0.001, determined by Student’s t-test

Growing evidence suggests that reduced mitochondrial respiration can reverse acquired chemoresistance (Mukherjee et al. 2023). Therefore, we next examined WBP1’s potential in overcoming chemoresistance. Using two primary CRC chemotherapeutics, 5-fluorouracil (5FU) and oxaliplatin, we established and validated resistant cell models to investigate whether WBP1 targeting could restore drug sensitivity. The resistant cell lines exhibited reduced sensitivity to 5FU (Fig. 6A) and oxaliplatin (Fig. 6B) compared to the parental cells. Interestingly, WBP1 knockout significantly sensitized 5FU-R (Fig. 6C) and oxaliplatin-R (Fig. 6D) cells to 5FU and oxaliplatin treatment, respectively.

Moreover, WBP1 knockout in the resistant cell lines also reduced mitochondrial respiration, as evidenced by the decreased basal, ATP-linked, and maximal OCR in 5FU-R (Fig. 6E and F) and oxaliplatin-R (Fig. 6G and H) cells. Additionally, WBP1 knockout increased the levels of lipid peroxidation (LPO) (Fig. 6I) and iron (Fig. 6J), indicating elevated ferroptosis.

Consistently, treatment with the mitochondrial function inhibitor rotenone also sensitized 5FU-R (Fig. 6K) and oxaliplatin-R (Fig. 6L) cells to 5FU and oxaliplatin, respectively. These results suggest that targeting WBP1 and its mediated mitochondrial respiration can reverse the acquired chemoresistance in CRC cells by inducing ferroptosis.

### High WBP1 expression correlates with chemoresistance-related genes and predicts poor survival in chemotherapy-treated CRC patients

**Fig. 7:**
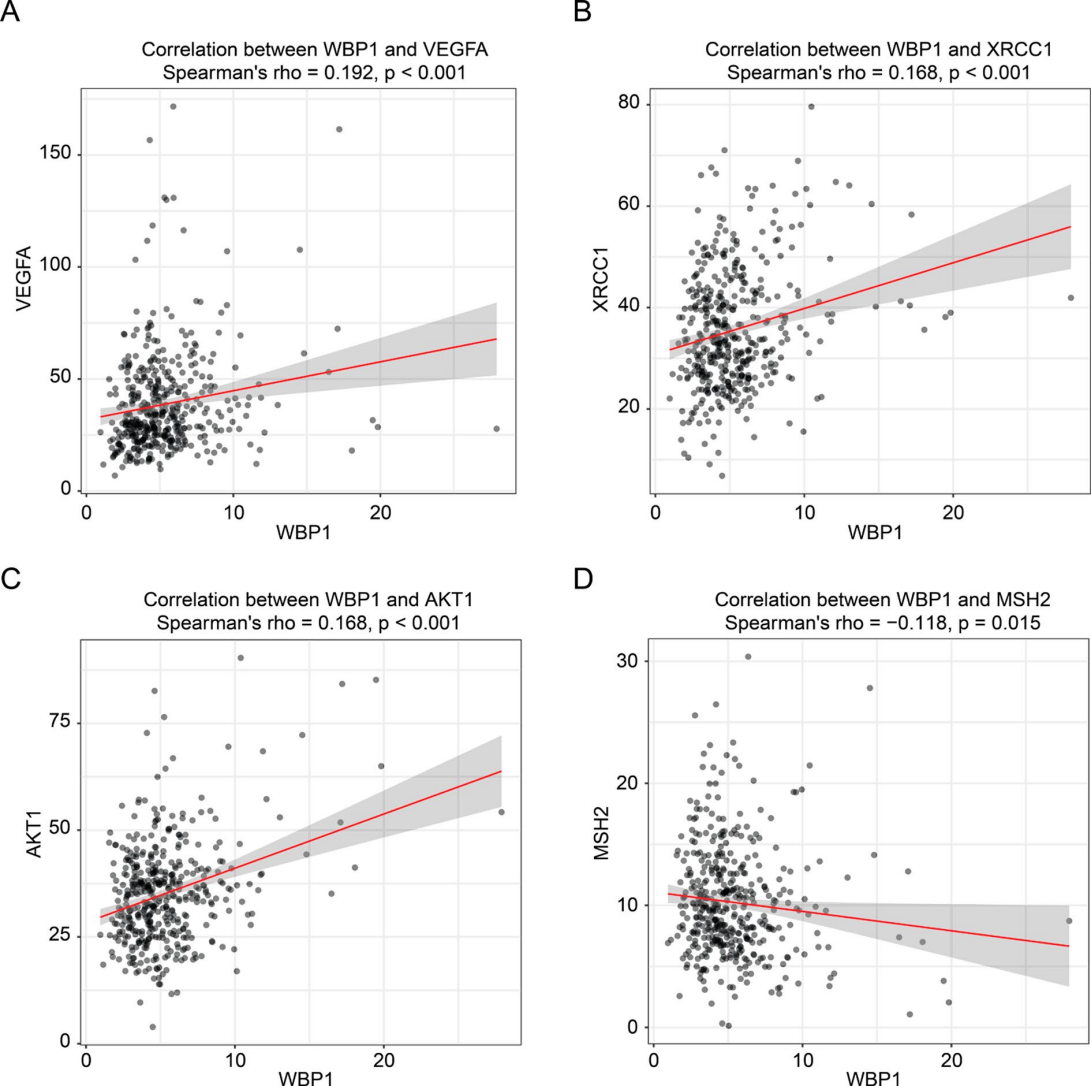
Correlation between WBP1 expression and chemoresistance-related genes in CRC patients. (**A**-**C**) Correlation between WBP1 expression and chemoresistance-promoting genes VEGFA (**A**), XRCC1 (**B**), and AKT1 (**C**) in CRC tissues from TCGA dataset (TCGA-COAD). (**D**) Correlation between WBP1 expression and chemosensitivity suppressor MSH2 in CRC tissues from TCGA dataset (TCGA-COAD). Spearman’s correlation coefficient (rho) and p-values are shown for each correlation analysis

To explore the clinical relevance of WBP1 in chemoresistance, we first analyzed the expression correlation between WBP1 and established chemoresistance-related genes using TCGA data. Spearman correlation analysis revealed that WBP1 expression positively correlated with several chemoresistance-promoting genes, including VEGFA (Li et al. 2020; Wei et al. 2022), XRCC1 (Stoehlmacher et al. 2001; Zhang et al. 2015b), and AKT1 (Golbashirzadeh et al. 2023; Pelullo et al. 2022) (Fig. 7A-C). In contrast, WBP1 showed negative correlation with MSH2, which suppresses chemoresistance by maintaining DNA mismatch repair function (Goodspeed et al. 2019) (Fig. 7D). These correlation patterns suggest that WBP1 may be functionally linked to known chemoresistance pathways in CRC.

**Fig. 8:**
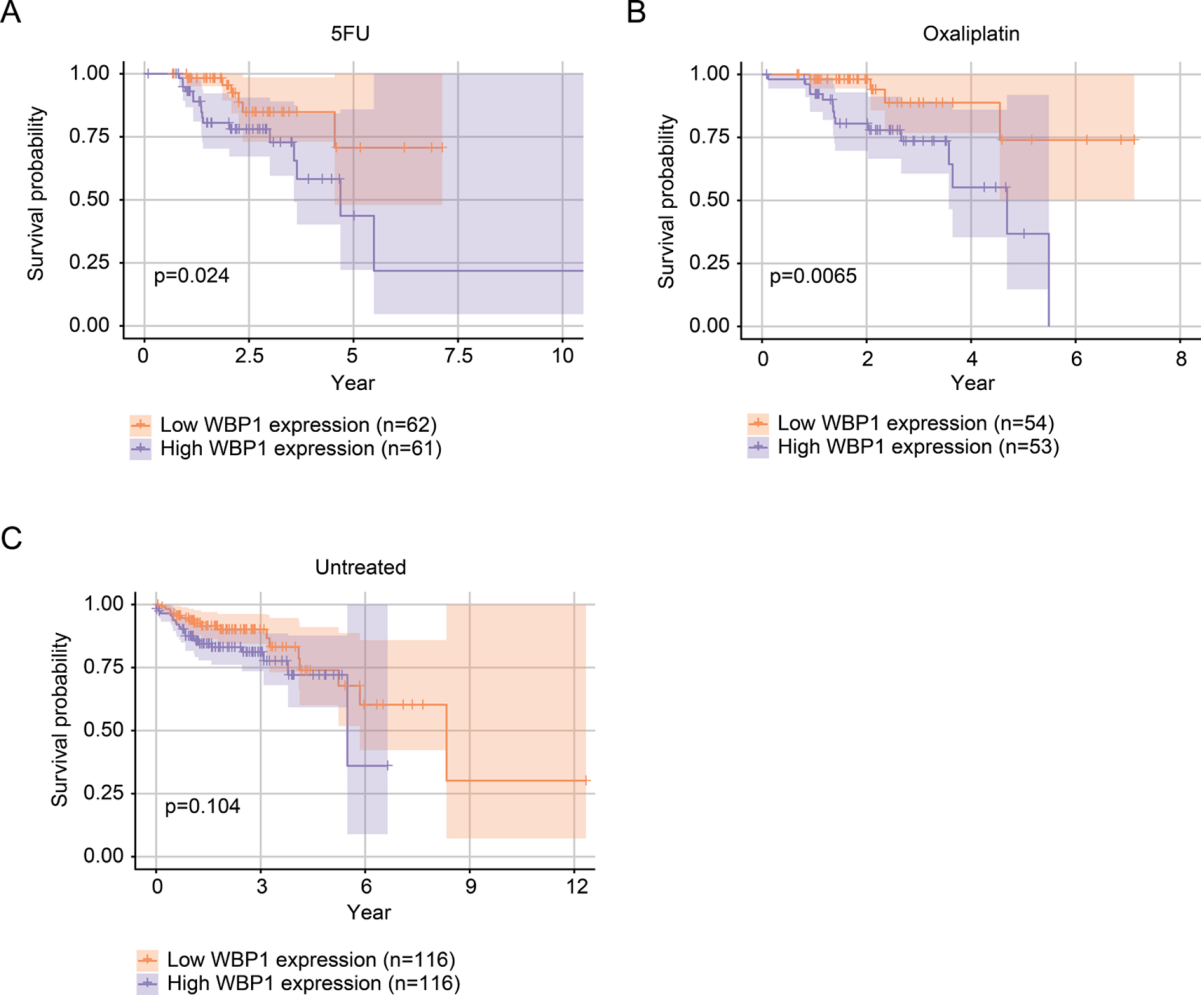
Elevated WBP1 expression predicts poor survival in CRC patients under chemotherapy treatment. (**A**-**C**) Overall survival probability was analyzed using Kaplan-Meier methods in CRC patients who received 5FU (**A**), oxaliplatin (**B**), or those not undergoing chemotherapy (**C**), categorized by WBP1 expression levels (either low or high) using median WBP1 expression as the threshold. The log-rank test was utilized to compute p-values. The data were sourced from public databases, with p-values below 0.05 considered statistically significant

Given these molecular associations, we next investigated whether WBP1 expression levels could predict clinical outcomes in chemotherapy-treated CRC patients. Kaplan-Meier analysis showed that elevated WBP1 levels were significantly linked to reduced overall survival in patients receiving 5FU (Fig. 8A) or oxaliplatin (Fig. 8B). In contrast, WBP1 expression had no significant impact on the survival of CRC patients without chemotherapy treatment (Fig. 8C). The adverse survival outcomes associated with high WBP1 expression in CRC patients under chemotherapy treatment further validates its involvement in chemoresistance mechanisms.

In conclusion, our findings show that WBP1 is essential for controlling mitochondrial respiration, cell growth, and ferroptosis in CRC cells. Targeting WBP1 or its mediated mitochondrial function can sensitize chemoresistant CRC cells to 5FU and oxaliplatin by inducing ferroptosis. The clinical significance is supported by TCGA data, linking elevated WBP1 to poor prognosis in CRC patients undergoing chemotherapy. Our work presents WBP1 targeting as a novel strategy to reverse chemoresistance by promoting ferroptotic cell death.

## Discussion

In this study, we explored the function of WBP1 in modulating mitochondrial activity, cell growth, and ferroptosis in CRC cells. Our findings reveal novel molecular mechanisms underlying CRC drug resistance and highlight WBP1’s potential as a therapeutic target for chemoresistant disease.

A key strength of our study lies in the comprehensive strategy we used to examine the role of WBP1 in CRC. We used a combination of genetic and pharmacological methods to manipulate WBP1 expression and mitochondrial function, which allowed us to establish a causal relationship between WBP1, mitochondrial respiration, and ferroptosis. The clinical significance of our findings was confirmed through TCGA database analysis, which showed WBP1’s effect on clinical outcomes in CRC patients receiving chemotherapy, pointing to its potential as a therapeutic target for improving treatment outcomes.

The selection of appropriate cellular models is crucial for ensuring the reliability and clinical relevance of experimental findings. In this study, we chose HCT116 and SW480 cell lines as they represent different molecular subtypes of CRC (MSI and MSS, respectively) and harbor distinct p53 status (wild-type versus mutant). This strategic selection allowed us to demonstrate that the role of WBP1 in regulating mitochondrial function and ferroptosis is conserved across different molecular backgrounds, suggesting a fundamental mechanism rather than a cell line-specific effect. The consistent results obtained from both cell lines, despite their distinct molecular characteristics, strengthen the validity of our findings and their potential clinical applicability. Furthermore, these cell lines are well-established models for studying chemoresistance in CRC, with documented responses to standard chemotherapeutic agents such as 5FU and oxaliplatin, making them particularly suitable for investigating the relationship between WBP1, mitochondrial function, and chemoresistance.

Our study also sheds light on the complex interplay between mitochondrial function, ferroptosis, and chemoresistance in CRC. While previous studies have suggested that mitochondrial dysfunction can promote ferroptosis and sensitize cancer cells to chemotherapy, our findings reveal that enhanced mitochondrial respiration, mediated by WBP1, can confer resistance to ferroptosis and chemotherapeutic agents in CRC cells. The data illustrate how mitochondrial functions vary across cancer contexts, emphasizing the importance of characterizing type-specific molecular pathways in chemoresistance.

Another novel aspect of our study is the identification of WBP1 as a regulator of mitochondrial function and ferroptosis in CRC. While WBP1 has been implicated in various cellular processes, its role in mitochondrial respiration and ferroptosis has not been previously explored. Our findings expand the current understanding of WBP1 function and suggest that it may play a broader role in cancer biology beyond its known involvement in cell cycle regulation and protein-protein interactions.

The tissue-specific nature of molecular mechanisms in cancer biology is increasingly recognized as crucial for developing targeted therapeutic strategies. Our comprehensive analysis of TCGA data across multiple cancer types revealed an intriguing pattern: the correlation between WBP1 expression and chemotherapy outcomes appears to be specific to CRC, as similar correlations were not observed in other cancer types (data not shown). This tissue specificity suggests that WBP1 might serve as a CRC-specific modulator of chemoresistance, potentially through unique interactions with CRC-specific molecular pathways or cellular contexts. Such specificity could be advantageous from a therapeutic perspective, as targeting WBP1 might provide a more focused approach for overcoming chemoresistance in CRC while potentially minimizing effects on other tissues. However, it is important to note that additional experimental validation across different cancer types would be valuable to fully understand the extent of WBP1’s tissue-specific effects. Future studies investigating the molecular basis of this specificity could provide valuable insights into both the fundamental biology of chemoresistance and the development of more effective targeted therapies for CRC.

Despite the strengths of our study, there are several limitations that should be acknowledged. First, our results are derived from in vitro experiments conducted on CRC cell lines, and further validation in animal models and primary tumor samples is necessary to confirm the role of WBP1 in chemoresistance and ferroptosis in vivo. Second, While our findings link WBP1 levels to survival in chemotherapy-treated CRC patients, prospective clinical studies are needed to evaluate the potential of using WBP1 as a biomarker to predict chemotherapy response and guide treatment decisions.

In conclusion, we identify WBP1 as an important regulator of mitochondrial function, cellular proliferation, and ferroptosis in CRC cells, offering a new therapeutic strategy to combat chemoresistance. These findings provide a foundation for future studies to further elucidate the molecular mechanisms underlying WBP1 function and to develop WBP1-targeted therapies for CRC treatment. Moreover, our results highlight the importance of investigating the role of mitochondrial function and ferroptosis in chemoresistance and suggest that targeting these processes may be a valuable approach to enhance cancer treatment outcomes.

Subsequent research should prioritize validating the role of WBP1 in chemoresistance and ferroptosis using animal models. The establishment of xenograft models using WBP1 KO and overexpressing CRC cells would provide valuable insights into whether WBP1 modulation affects tumor growth, chemotherapy response, and ferroptosis sensitivity in vivo. Critical experimental endpoints would include tumor growth kinetics, mitochondrial function analysis in tumor tissues, and assessment of ferroptosis markers. Furthermore, patient-derived xenografts would offer a more clinically relevant platform to validate these findings. The technical challenges inherent in such in vivo studies, particularly maintaining consistent drug delivery and measuring ferroptosis markers, could be addressed through systematic optimization of experimental protocols.

The molecular mechanisms by which WBP1 regulates mitochondrial function and ferroptosis should be further explored to identify potential downstream targets and signaling pathways. This would require both detailed in vitro mechanistic studies and subsequent validation in animal models. Additionally, the development of specific WBP1 inhibitors and their evaluation in combination with chemotherapeutic agents in preclinical and clinical settings could lead to novel therapeutic strategies for overcoming chemoresistance in CRC patients. The completion of these proposed animal studies would provide crucial data to guide the development of WBP1-targeted therapies.

## Electronic supplementary material

Below is the link to the electronic supplementary material.

Supplementary Material 1

## Data Availability

Transcriptomic data for normal colorectal tissues and CRC samples were obtained from The Cancer Genome Atlas (TCGA) database under the project identifier TCGA-COAD. This dataset is publicly available, and researchers can access and download it for further analysis through the TCGA Data Portal.
