# Supplementary material for: WBP1 regulates mitochondrial function and ferroptosis to modulate chemoresistance in colorectal cancer
Source: Mol Med. 2025 Mar 12;31:93. doi: 10.1186/s10020-025-01151-3 (PMC11900258; doi:10.1186/s10020-025-01151-3)

## Figure S1. WBP1 depletion leads to decreased levels of markers associated with mitochondrial function in CRC.

(A-B) Real-time PCR analysis of mitochondrial function related markers (MT-ND1 and MT-CO1) and WBP1 in WT and WBP1 KO HCT116 (A) and SW480 (B) cells. Data are presented as mean + SD. *P < 0.05, **P < 0.01, ***P < 0.001 versus WT by Student's t-test. (C-D) Western blot analysis of MT-ND1, MT-CO1, and WBP1 protein levels in WT and WBP1 KO HCT116 (C) and SW480 (D) cells. TUBA1A served as a loading control.


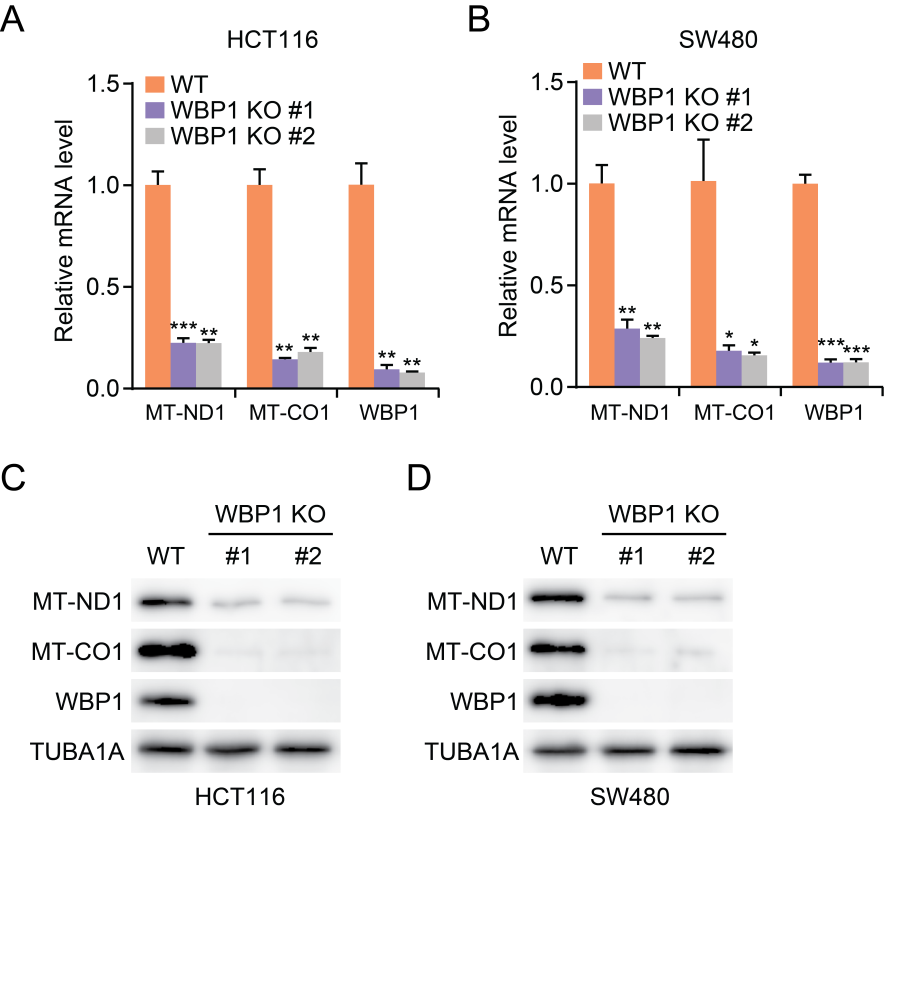


## Figure S2. WBP1 depletion reduces the expression of ferroptosis defense markers in CRC cells.

(A-B) Real-time PCR analysis of key ferroptosis suppressors (GPX4 and FTH1) and WBP1 in WT and WBP1 KO HCT116 (A) and SW480 (B) cells. Data are presented as mean + SD. *P < 0.05, **P < 0.01 versus WT by Student's t-test. (C-D) Western blot analysis of GPX4, FTH1, and WBP1 protein levels in WT and WBP1 KO HCT116 (C) and SW480 (D) cells. TUBA1A served as a loading control.


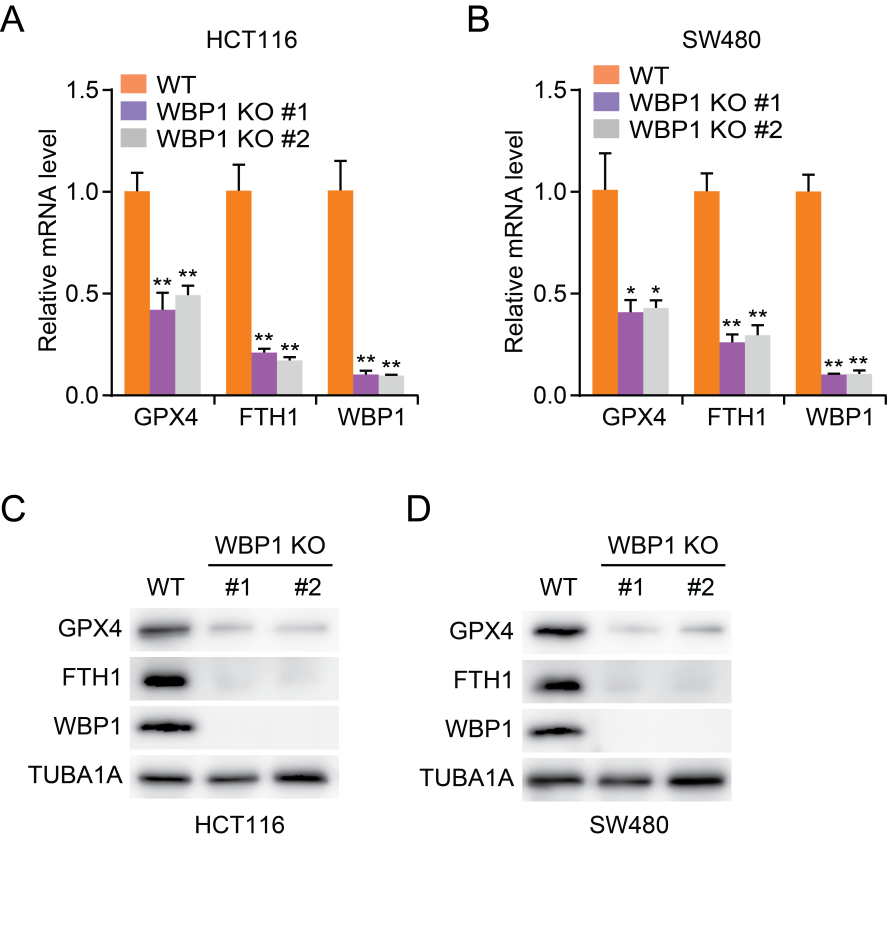


## Figure S3. WBP1 protein levels in rotenone-treated and WBP1-overexpressing CRC cells.

(A-B) Western blot analysis of WBP1 protein levels in HCT116 (A) and SW480 (B) cells treated with DMSO, rotenone (100 nM for HCT116 and 200 nM for SW480), or rotenone in WBP1-overexpressing cells.


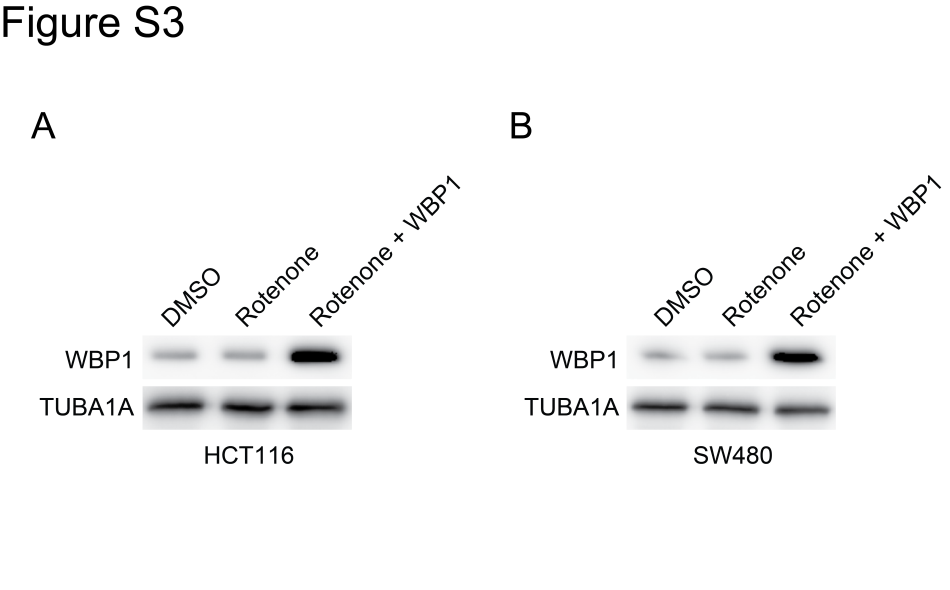

Supplement: Supplementary file 1 — Supplementary Material 1 [file 10020_2025_1151_MOESM1_ESM.docx]
